# Determinants of delay in timely treatment seeking for diarrheal diseases among mothers with under-five children in central Ethiopia: A case control study

**DOI:** 10.1371/journal.pone.0193035

**Published:** 2018-03-27

**Authors:** Guteta Degefa, Measho Gebreslassie, Kidanu Gebrameriam Meles, Ruth Jackson

**Affiliations:** 1 Department of Epidemiology, College of Health Sciences, Mekelle University, Mekelle, Ethiopia; 2 Department of Health System, College of Health Sciences, Mekelle University, Mekelle, Ethiopia; 3 Alfred Deakin Institute, Deakin University, Geelong, Australia; University of Washington, UNITED STATES

## Abstract

**Background:**

Delays in seeking timely appropriate care contributes to a large number of deaths from diarrhea in children. This study aimed to identify determinants of delays in seeking timely treatment by mothers/caregivers of under-five children with diarrheal diseases.

**Methods:**

We used an unmatched case-control study from February—March 2017 among 316 children: 158 cases and 158 controls. Cases were mothers/caregivers with under-five children who had signs/symptoms of diarrhea and sought treatment after 24 hours onset of symptom. Controls sought treatment within 24 hours. Field workers collected data using a pre-tested standardized questionnaire. Multivariate logistic regression was conducted to identify determinants of delay in timely diarrhea treatment seeking. Statistical significance was declared by using a p-value<0.05 and 95% of confidence interval (CI) for an adjusted-odds ratio (AOR).

**Results:**

The determinants of delay in timely treatment seeking of mothers/caregivers of under-five children with diarrheal diseases were children <24months (AOR = 1.9,95%CI:1.1–3.4); fail to attend school (AOR = 2.4, 95%CI:1.2–4.6); being female children (AOR = 1.7,95%CI:1.05–2.9); preferring government health facility for the treatment of children with diarrheal diseases (AOR = 2.9, 95%CI, 1.3–6.7); lack of past history taking children to health facility and lack of counseling (AOR = 4.8, 95%CI:2.0–12.1); being in the15-25 years age (AOR = 1.7, 95%CI:1.1–3.0) and taking children to a health facility as a first response to diarrhea (AOR = 0.1, 95%CI:0.01–0.8).

**Conclusions:**

Age of the child, maternal age, and disease related determinants were determinants for seeking timely treatment to diarrheal diseases. Providing skilled based health education and counseling to mothers/caregivers on seeking timely treatment and taking children with diarrheal diseases to a health facility as a first response to diarrhea is a paramount intervention to reduce morbidity and mortality of children.

## Introduction

Diarrheal disease is one of the five leading causes of morbidity and mortality among under-five children in the world [1]. Approximately, 500, 000 under-five children die from diarrhea each year and more than 2,200 children die every day [2–4]. The World Health Organization (WHO) estimated that African and South East Asia Regions jointly shared 78% of all diarrhea deaths occurring among children in the developing countries [5]. In sub-Saharan Africa, where high rates of child mortality were reported, one in eight children dies before the age of five [5]. According to 2014 WHO estimates, diarrhea contributed 9% to the total under-five children mortality in Ethiopia [6]. Moreover, the Ethiopian Demographic and Health Surveys (EDHS) of 2011 and 2016 reported that 13% and 12% of under-five children respectively were reported to have had diarrhea two weeks before the survey [7, 8]. Furthermore, morbidity reports and different community-based studies have also publicized that the two-week prevalence of diarrhea varies from 19.6% to 33.2%; showing that diarrheal disease is a major public health problem that causes high morbidity and mortality of children in Ethiopia [9, 10, 11].

A large number of children in low-income countries die due to a delay in seeking treatment timely from health care [9, 12]. In Ethiopia, health care seeking is poor and only a small proportion of children receive appropriate treatment timely [10]. Nationally, only 32% of under-five children with diarrheal diseases were taken for advice or treatment to a health facility [10]. Even though different studies identified low health seeking for under-five children with diarrheal diseases, there are few studies regarding determinants of delays in timely treatment seeking [13, 14]. Up-to-date evidence on status for determinant factors associated with timely treatment seeking of mothers/caregivers of under-five children with diarrheal diseases in Ethiopia is essential.

## Methods

### Study design, period and area

We used an unmatched case-control study from February-March, 2017. The study was conducted in Woliso district, which is one of the Zonal towns in the Oromia Region, located 114 km southwest of Addis Ababa. Clean water coverage in the town was above 98% while the coverage of standardized latrine was 94.4% [14]. Childhood diarrhea was one of the leading top ten diseases in under-five clinics in Woliso town [15].

### Source and study population

We obtained a sample from all under-five year children living in Woliso town and surrounding rural kebeles (the lowest administrative unit) who visited under-five clinics with diarrheal diseases. Cases were under-five children with signs/symptoms of diarrhea whose mothers sought treatment after 24-hours of the recognition of diarrhea. Controls were under-five children with signs/symptoms of diarrhea whose mothers sought treatment within 24-hours of the recognition of diarrhea. Children whose mothers/caregivers did not mention the exact dates of onset of diarrhea were excluded.

### Sample size and sampling procedure

We calculated sample size using two-population proportion-formula by assuming odds ratio of 2[16], 95%CI, 80% power and 1:1 case to control ratio. Adding five percent maximum acceptable difference and five percent nonresponse rate, the total sample size was 316 children (158 cases: 158 controls). Three government and two private health facilities were included by simple random sampling technique.

We selected respondents by systematic random sampling techniques. Starting from the first participant selected by random sampling procedures (1 to K^th^ = 2,3 by lottery method) from the first interval, every K^th^ individuals at the succeeding intervals were selected separately for cases and controls proportionally based on the number of diarrhea cases reported in February 2017 Ethiopian fiscal year (EFY), same month, of data collection from each health facility. Proportional allocation of the respondents to the five health facilities was conducted. When mothers/caregivers complained of diarrhea in their child completed their consultation with a health professional, they moved to a private room for an interview until the total required sample size obtained.

### Data collection procedure

Field workers collected data using a structured questionnaire to assess predisposing, enabling, disease-related factors and promptness of treatment seeking for childhood diarrhea. We used the patient history card to take vital signs and diarrhea related complications. Data were collected under the follow-up of the trained supervisors. Cases and controls were recruited from hospital and health centers/clinics after they came to the facilities and were diagnosed with diarrhea.

### Data quality control

We used a pretested structured and standardized questionnaire adopted from EDHS [10]. The questionnaire was initially prepared in English(S1 File) and translated into Afan-Oromo, the local language by a language expert. We gave two days training for data collectors and supervisors. We conducted a pre-test on five percent of the total sample size before the actual data collection in Adama health center. Based on the result of pre-testing, necessary revision like timing, skipping pattern and coherence was conducted.

### Data processing and analysis

Data-clerks entered the data into Epi-Info version 7.1.5. Data were analyzed using SPSS version 20.0. Bivariate and multivariate logistic regression analysis were conducted to assess determinants of delay in timely diarrhea treatment seeking of mothers/caregivers of under-five children with diarrheal diseases at a P-value<0.05, 95% confidence interval and odds ratio (S2 File).

### Operational definition

#### Field workers

Data collectors who have experience in data collection of surveys.

#### Caregiver

Any person above 18 years of age who at the time of the study was directly responsible for the care of the child.

#### Care seeking

Any care sought from defined governmental or nongovernmental health facility for a child with diarrheal diseases.

#### Treatment delay

Care that was sought from health facilities after 24 hours from the recognition of the presence of diarrhea in under-five children.

#### Timely treatment seeking

Care that was sought from health facilities within 24 hours from the recognition of the presence of diarrhea in under-five children.

### Ethical considerations

We obtained informed verbal consent from mothers/caregivers because the study was not a sensitive issue and it has no any harm and procedure. We secured permission letter from Oromia Regional Health Bureau. Data collection was conducted confidentially and data de-identified, de-linked and stored in a secure location. Informed verbal consent was obtained from each subject so that the study could be published and presented at different workshops while protecting the participants’ confidentiality. The informed verbal consent procedure was approved by Institutional Review Board of Mekelle University College of Health Sciences. Finally, we obtained ethical clearance from the Institutional Review Board of Mekelle University College of Health Sciences (Reference letter ERC0959/2017).

## Results

### Child and parental characteristics

In our study, 316 mothers (158 cases: 158 controls) were interviewed, making a response rate of 100%. The majority, 120 (75.9%) of the cases and 95(60.1%) controls were children <24-months. The mean age of cases was 18.7(±12.5SD) and that of controls was 19.4(±11.6SD) months. More than half of respondents were females, 85(53.8%) among cases and males, 94(59.5%) among controls (**Table 1**).

**Table 1:** Predisposing factors of delay in timely treatment seeking for mothers/caregivers of <5 children with diarrheal diseases in Woliso town, central Ethiopia, 2017 (n = 316).

Variables	Patient category	
	Cases (n = 158) No (%)	Controls (n = 158) No (%)
**Age of child in months**		
< 24	120(75.9)	95(60.1)
≥ 24	38(24.1)	63(39.9)
**Sex of child**		
Male	73(46.2)	94(59.5)
**Birth order of child**		
≤ 2	104(65.8)	108(68.4)
3–5	49(31.0)	44(27.8)
≥5	5(3.2)	6(3.8)
**Age category of mothers/caregivers**		
15–25	61(38.6)	57(36.1)
26–34	86(54.4)	91(57.6)
≥ 35	11(7.0)	10(6.3)
**Ethnicity of Mother/caregiver**		
Oromo	112(70.9)	117(74.1)
Amhara	20(12.7)	19(12.0)
Gurage	26(16.5)	22(13.9)
**Religion of Mother/caregiver**		
Orthodox	71(44.9)	76(48.1)
Protestant	60(38.0)	54(34.2)
Muslim	27(17.1)	28(17.7)
**Marital status of Mother/caregiver**		
Single	0(0.0)	3(1.9)
Married	151(95.6)	151(95.6)
Divorced	7(4.5)	4(2.6)
**Place of residence**		
Rural	65(41.1)	43(27.2)
Urban	93(58.9)	115(72.8)
**Educational level of Mother/caregiver**		
No education	61(38.6)	27(17.1)
Primary	56(35.4)	70(44.3)
Secondary	27(17.1)	44(27.8)
More than secondary	14(8.9)	17(10.8)
**Educational level of Father**		
No education	18(11.4)	11(7.0)
Primary	77(48.7)	56(35.4)
Secondary	39(24.7)	61(38.6)
More than secondary	24(15.2)	30(19.0)

More than 1/4 61(38.6%) cases and 57(36.1%) control mothers/caregivers were between 15–25 years of age. Three-fourths 112(70.9%) cases and 117(74.1%) controls were among the member of the Oromo ethnicity and 147(46.5%) were Orthodox Christian. More than three-fourths of cases 151(95.6%) and controls 151(95.6%) were married. More than 1/4 61(38.6%) cases and 27(17.1%) controls had no education while 77(48.7%) cases and 56(35.4%) controls fathers attended primary education (**Table 1**).

#### Enabling factors

Almost half 71(44.9%) of cases and 89(56.3%) controls were housewives while 49(31.0%) cases’ and 32(20.3%) controls’ father were farmers. The majority, 148(93.7%) of cases and 130(82.3%) controls preferred government health facilities. The cost of treatment 258(81.6%), examination of child 159(50.5%) and distance to health facilities 122(38.6%) were the major reasons for the selection of health facilities (**Table 2**).

**Table 2:** Enabling factors of timely treatment seeking of mothers/caregivers of <5 children with diarrheal diseases in Woliso town, Ethiopia, 2017(n = 316).

Variables	Category	
	Cases (n = 158) No (%)	Controls (n = 158) No (%)
**Occupation of the mothers/caregivers**		
Housewife	71(44.9)	89(56.3)
Government worker	11(7.0)	22(13.9)
Merchant	27(17.1)	35(22.2)
Farmer	32(20.3)	6(3.8)
Labor worker	1710.8)	6(3.8)
**Occupation of Father**		
Government worker	26(16.5)	34(21.5)
Merchant	38(24.1)	65(41.1)
Farmer	49(31.0)	32(20.3)
Labor worker	33(20.9)	10(6.3)
Student	3(1.9)	0(0.0)
Others	9(5.7)	17(10.8)
**Monthly income**		
≤ 1250	41(25.0)	26(16.4)
1251–2500	73(46.2)	51(32.3)
≥ 2501	44(27.8)	81(51.3)
**Cost of treatment**		
Easy to pay	87(55.1)	100(63.3)
Difficult to pay	68(43.0)	56(35.4)
Very difficult to pay	3(1.9)	2(1.3)
**Nearby health facility to the family**		
Government	137(86.7)	133(84.2)
Private	21(13.3)	25(15.8)
**Preferred health facility**		
Government	148(93.7)	130(82.3)
Private	10(6.3)	28(17.7)
**Reason for selecting gov’t health facility**		
Do not charge too much	140(88.6)	118(74.7)
Nearness	75(47.5)	47(29.7)
Respect given	5(3.2)	5(3.2)
Examination given	74(46.8)	52(32.9)
Low waiting time	2(1.3)	1(0.6)
Treatment is effective	7(4.4)	17(10.8)
Always open	0(0.0)	11(7.0)
**Reason for selecting private health facility**		
Respect given	3(1.9)	6(3.8)
Examination given	9(5.7)	24(15.2)
Low waiting time	6(3.8)	20(12.6)
Treatment is effective	2(1.3)	7(4.4)

#### Need or disease-related factors

Most of the mothers/caregivers 303(95.9%), responded to diarrhea of children by taking them to the health facility. Roughly 80(50.6%) of cases and 102(64.6%) controls took their children immediately when diarrhea of children was complicated with vomiting, 82(51.9) of cases and 85(53.8) controls due to inability to feed, and fever for 81(51.3%) cases and 82(51.9%) of controls. Thirty-five (22.2%) of cases and 53(33.5%) controls mothers/caregivers reported that they seek medical care for any diarrhea. With current diarrhea, 134(42.4%) of respondents complained of vomiting, 92(29.1%), unable to feed and 143(45.3%) had fever. Twenty (12.6%) of cases and 9(5.7%) controls mothers/caregivers believe that diarrhea can be cured by itself. Aproximatly 25(15.8%) of cases and 40(25.3%) controls developed diarrhea within the last six months and visited a health facility (**Table 3**).

**Table 3:** Need/disease-related factors of timely treatment seeking of mothers/caregivers of <5 children with diarrheal diseases in Woliso town, Ethiopia, 2017 (n = 316).

	Category	
Variables	Cases (n = 158)	Controls(n = 158)
	N (%)	N (%)
**First response to child diarrhea**		
Take to health facility	146(92.4)	157(99.4)
Take to traditional healer	3(1.9)	0(0.0)
Treat with drug from Pharmacy	9(5.7)	0(0.0)
**Symptoms of diarrheal disease**		
Diarrhea	21(13.3)	23(14.6)
Blood in diarrhea	97(61.4)	85(53.8)
Vomiting	89(56.3)	83(52.5)
Unable to feed	82(51.9)	85(53.8)
Fever	6(3.8)	14(8.9)
Sunken Eye-ball	26(16.5)	36(22.8)
**Symptoms associated with current diarrhea**		
Blood in diarrhea	12(7.6)	14(8.9)
Vomiting	80(50.6)	102(64.6)
Unable to feed	50(31.6)	42(26.6)
Fever	75(47.5)	68(43.0)
Increased thirsty	14(8.9)	8(5.1)
Irritability	13(8.23)	8(5.1)
Increased frequency of diarrhea	35(22.2)	53(33.5)
Only diarrhea	4(2.5)	2(1.3)
**Think diarrhea cured by itself**		
No	138(87.3)	149(94.3)
**Think diarrhea harm children**		
Yes	147(93.0)	157(99.4)
**Effect of diarrhea on children**		
Make child weak and malnourished	15(9.5)	14(8.9)
Kill the child	39(24.7)	25(15.8)
Make weak and kill	87(55.1)	105(66.4)
**Child develop diarrhea since six month**		
Yes	25(15.8)	40(25.3)
**Visit health facility before for diarrhea**		
Yes	25(15.8)	40(25.3)
**Visit in the six-month help for the current visit**		
Yes	16(10.1)	38(24.1)
**How to visit in the last 6-month help for current visit**		
Counseled importance of visiting health facility	7(4.4)	31(19.6)
Satisfied with treatment given	10(6.3)	7(4.4)
Others	8(5.1)	2(1.3)
**Know child died of diarrhea**		
No	155(98.1)	154(97.5)
**Dehydration status**		
Yes	16(10.1)	11(7.0)

### Determinants of delay in timely treatment seeking

Binary and multivariate logistic regression presented with odds ratio and 95%CI for the variables that predict timely treatment seeking of mothers/caregivers of under-five children with diarrhea (**Table 4**). Multivariate logistic regression shows that sex and age of child, mothers/caregivers’ age, school attendance, preference of health facility, taking children to health facility as first response to diarrhea and previous counseling about the importance of timely visits to a health facility were associated with delays in treatment seeking of mothers/caregivers of under-five children with diarrheal diseases. Mothers/caregivers of female children had higher odds of experiencing a delay compared with mothers/caregivers of male children (AOR = 1.7,95%CI:1.1–2.9). Mothers/caregivers of younger children with diarrheal diseases were two times more likely to delay to seek treatment compared with mothers/caregivers of older children (AOR = 1.9,95%CI:1.10–3.4).

**Table 4:** Determinants of delay in timely treatment seeking of mothers/caregivers of <5-children with diarrhea in Woliso town, Ethiopia, 2017 (n = 316).

Variables	Cases	Controls	COR(95%CI)	AOR(95%CI)
	No (%)	No (%)		
**Sex**				
Male	73	94	1	1
Female	85	64	1.7(1.1,2.7)	1.74(1.1,2.9)^*^
**Child age category in months**				
< 24	120	95	2.1(1.3,3.4)	1.91(1.10,3.4)^*^
≥ 24	38	63	1	1
**Mother age category in years**				
15–25	64	50	1.7(1.1,2.7)	1.7(1.1,3.0)^*^
26–35	75	98	0.7(0.3,1.6)	0.9(0.4,2.5)
≥ 36	19	10	1	1
**Attend school**				
No	61	27	3.0(1.8,5.1)	2.4(1.2,4.6)^*^
Yes	97	131	1	1
**Monthly income**				
≤ 1250	41	26	2.9(1.6,5.4)	0.97(0.8,2)
1251–2500	73	51	1.1(0.6,2.0)	1.8(0.9,3.8)
≥ 2501	44	81	1	1
**Preferred Health facility**				
Gov’t	148	130	3.2(1.5,6.8)	2.9 (1.3,6.7)^*^
Private	10	28	1	1
**Perceived cost at the preferred health facility**				
High	40	18	2.6(1.5,5)	0.6(0.7,4.0)
Low	118	140	1	1
**Perceived distance of the preferred Health facility**				
Far	75	47	2.1(1.4,3.4)	0.9(0.5,1.6)
Near	83	111	1	1
**Waiting time at preferred health facility**				
High	150	137	2.9(1.3,4)	0.9(0.2,4.7)
Low	8	21	1	1
**Necessary medication available**				
No	149	134	3.0(1.3,6.6)	1.6(0. 7,4.0)
Yes	9	24	1	1
**Visit health facility as first response to any diarrhea**				
No	12	1	1	1
Yes	146	157	0.1(0.01,0.7)	0.1(0.01,0.8)^*^
**Has only diarrhea**				
No	35	54	1	1
Yes	123	104	1.8(1.1,2.9)	0.9(0.4,2.2)
**Develop diarrhea in the last 6-months**				
No	133	118	1	1
Yes	25	40	0.6(0.3,0.9)	1.1(0.4,3.3)
**Visit health facility for diarrhea in the last 6-month**				
No	133	118	1.8(1.03,3.2)	0.7(0.3,1.8)
Yes	25	40	1	1
**Did visit in the last six-month help for the current visit**				
No	142	120	2.8(1.4,5)	0.3(0.05,1.6)
Yes	16	38	1	1
**Counseled importance of timely visiting health facility**				
No	151	127	5.3(2.2,12.3)	4.8(2.0,12.1)^*^
Yes	7	31	1	1

*Significant by both bivariate and multivariate logistic regression at p-value of < 0.05

Mothers 15–25 years of age were more likely to delay to seek timely treatment (AOR = 1.7,95%CI:1.1–3.0) for <5-children with diarrhea than mothers/caregivers between 26–35 years age. Mothers/caregivers who did not attend school were more likely to delay seeking treatment for under-five children with diarrhea than mothers/caregivers who attend school (AOR = 2.4,95%CI: 1.20–4.6).

Mothers/caregivers who preferred a government health facility for the treatment of children with diarrhea were three times more likely to delay to seek treatment compared with those preferred private health facilities (AOR = 2.9,95%CI:1.3–6.7). Mothers/caregivers who take their children to health facilities as a first response to any diarrhea were 90% less likely to delay to seek treatment for children with diarrhea (AOR = 0.1, 95%CI:0.01–0.8) compared to those did not believe in taking their children to a health facility. Mothers/caregivers who had no history of taking their children to a health facility and being counseled about the importance of timely treatment seeking for diarrhea of children were five times more likely to bring their under-five children late for the treatment of diarrhea than their counterparts (AOR = 4.8,95%CI:2.0–12.1) (**Table 4**).

## Discussion

Child sex and age, mothers/caregivers’ age, school attendance, preference of health facilities, the first response to diarrhea and counseling about the importance of timely visiting health facility were predictors of treatment seeking for <5-children with diarrheal diseases within and after 24-hours of symptom onset.

Mothers are less likely to seek treatment for girls than for boys. This finding is similar to EDHS report and a study conducted in Nepal that identified the sex of the child determines treatment seeking [17–19]. On the contrary, this finding differed from studies in Nigeria and India that reported sex had no association with mothers/caregivers’ treatment seeking for under-five children [20, 21]. The possible reason for the difference in seeking treatment differently for male and female children could be due to cultural influence and gender inequality that systematically disadvantages females in the community, which in turn, may impose mother/caregiver to give priority to a male child.

The age of the child associated with a doubled risk of delay in seeking treatment timely. This finding is supported by EDHS 2011 report and a study conducted in India [7, 21]. However, the finding was different from a study conducted in central Ethiopia, China and India that reported mothers/caregivers who had younger children less than 24-months were more likely to seek health care services than mothers/caregivers of older children [12,21, 22]. This variation may be due to believing that mothers/caregivers link diarrhea with the eruption of milk teeth that in younger children result in mild and self-limited diarrhea.

Maternal age was a determinant factor for timely treatment seeking for under-five children with diarrhea. This study is similar to study conducted in Ethiopia in 2015 and Kenya in 2012 that found better treatment seeking of mothers/caregivers of children when mothers/caregivers were in their twenties or early thirties [23, 24]. Mothers/caregivers in the younger age group may have lower incomes and less power to make healthier decisions regarding the health of their children than mothers/caregivers in their late twenties and early thirties. The other reasons might that they have had less experience dealing with the complications of diarrhea and may expect spontaneous recovery.

Educational status was also a determinant factor for mothers seeking treatment for their under-five children with diarrhea. This is consistent with studies from central Ethiopia and Yemen [9, 13, 25]. However, this is in contrary with a study conducted in Niger [26]. This variation may be mothers/caregivers who attended school are thought to have a better opportunity to learn health information than those who did not attend school. Moreover, illiterate mothers/caregivers may not have basic knowledge on the impacts of the potential risk of delay in seeking treatment timely.

Mothers/caregivers who were not counseled about the importance of timely treatment seeking for diarrhea of children at a health facility was a determinant factor. This is consistent with a study conducted in Ethiopia that identified the benefit of prior information for mothers/caregivers while visiting health facilities timely for childhood diarrhea [23].

Preference of health facility for treatment of children with diarrheal diseases was a predictor of delay to seek treatment. This is similar to a study conducted in low-income countries and found a high rate of consultation at private providers due to factors related to convenience, prompt care and more courteous service that determine treatment seeking in government health facilities [14]. However, this differs from a study done in Ethiopia which stated that government health facilities are sought for the treatment of children with diarrhea, since the costs are relatively cheap for the mothers/caregivers [27].

Household income and cost of a health facility were not determinant factors. This is similar to other studies that cited cost as a reason for not seeking care for children [28–30]. However, other studies in Ethiopia and Niger reported that household income and cost of health facility were predictors of health care seeking of mothers/caregivers of under-five children [17, 26]. The difference may be due to the capacity to pay for health facilities that may have less variation between these respondents, in which the majority report the costs of treatment was easy to pay.

## Conclusions

Age of children, mothers/caregivers age, school attendance, preference of health facilities, the first response to diarrhea and history of previous counseling about the importance of timely treatment seeking were determinants of delay to seek treatment timely within 24 hours of recognition of diarrhea in under-five children. Hence, preventive care programs should target age, preference of health facility, and taking children with diarrheal diseases to health facility early. Awareness creation among mothers/caregivers about the importance of timely treatment seeking to improve timely treatment seeking of under-five children with diarrheal diseases is essential.

## Supporting information

S1 File Engish version questionnaire used to collect raw data in central Ethiopia, 2017. (PDF)

S2 File Minimal raw data set in zip file,2017. (ZIP)

## Abbreviations

AOR: Adjusted odds ratio
CI: Confidence interval
EDHS: Ethiopia Demographic and Health Survey
EFY: Ethiopian Fiscal Year
WHO: World Health Organization
