# Supplementary material for: Determinants of delay in timely treatment seeking for diarrheal diseases among mothers with under-five children in central Ethiopia: A case control study
Source: PLoS One. 2018 Mar 27;13(3):e0193035. doi: 10.1371/journal.pone.0193035 (PMC5870934; doi:10.1371/journal.pone.0193035)
Supplement: S1 File — (PDF) [file pone.0193035.s001.pdf]

English version questionnaire used to collect data in Central Ethiopia

Section 1: General information about child and parental socio-demographic characteristics.

101	Age of child	------------- in Months	**Skip**
102	Age of mother/ caregiver during the interview	------------ in Years	
103	Sex of child	1. Male<br>2. Female	
104	Birth order of child	1. First 2. Second<br>3. Third 4. Fourth<br>5. Fifth and above	
105	Place of residence	1. Rural 2. Urban	
106	Marital status of mother/caregiver	1. Single 2. Married<br>3. Divorced 4. Widowed	
107	Religion of mothers/caregiver	1. Orthodox 2. Protestant<br>3. Muslim 4. Catholic<br>5. Other (specify)………	
108	Ethnicity	1. Oromo 2. Amhara<br>3. Gurage<br>4. Other (specify)……	
109	Education status of mother/care giver	1. Unable to read and write.<br>2. Read and write<br>3. Primary school<br>4. Secondary school<br>5. Certificate course<br>6. Diploma and above	
110	Is mother/caregiver graduated from health extension packages?	1. Yes<br>2. No	
111	Is mother/caregiver involved in community conversation?	1. Yes<br>2. No	
112	Education of father	1. Unable to read and write.<br>2. Read and write<br>3. Primary school<br>4. Secondary school<br>5. Certificate course<br>6. Diploma and above	
113	Occupation of mothers/caregivers	1. House wife's<br>2. Governmental work<br>3. Merchant<br>4. Farmer<br>5. Labor worker<br>6. Student<br>7. Other (specify)………	
114	Occupation of Father	1. Governmental work<br>2. Merchant<br>3. Farmer<br>4. Labor worker<br>5. Student<br>6. Other (specify)………..	
115	Number of family members	---------- in Numbers	

Section 2: Enabling factors

201	Monthly income	--------------- ETB/month	

202	Cost of treatment in health facility for child hood diarrhea?	1. Easy to pay<br>2. Difficult to pay<br>3. Very difficult to pay	
203	Does your house have the following material?	1. Radio 1. Yes 2. No<br>2. Television 1. Yes 2. No<br>3. Telephone 1. Yes 2. No<br>4. Electricity 1. Yes 2. No<br>5. Car or track 1.Yes 2. No	
204	Which health facility is close to your house?	1. Government<br>2. Private	
205	Which health facility do you prefer for children with diarrhea?	1. Government<br>2. Private	
206	Why did you prefer the selected health facility	1. They do not charge to much<br>2. Because, they are near<br>3. Because, they are respectful<br>4. Thorough examination<br>5. Low waiting time<br>6.Necessary medication is available<br>7. Treatment is effective<br>8. Always open or early opening<br>9. Other (Specify)……	
207	How close is the selected facility on foot?	1. < 15 minutes<br>2. 15-30 minutes<br>3. 30-60 minutes<br>4. One-Two hour<br>5. Above two hour	

Section 3: Disease related factors

301	What was your first response when your Child had got diarrhea?	1. Take to health facilities<br>2. Take to traditional treatment<br>3. Self-treatment at home<br>4. Treat the child with drug buying from pharmacies or drug sellers without prescriptions<br>5. Treat with holy water<br>6. Did nothing	
302	When did you sick medical care for diarrhea of children?	1. Blood in diarrhea<br>2. Child vomits every thing<br>3. Child unable to feed or feed poorly<br>4. Has fever<br>5. Eye ball sunken<br>6. For any diarrhea<br>7. Other (specify)……	

303	What makes you seek medical care today?	1. Blood in diarrhea<br>2. Child vomits every thing<br>3. Child unable to feed or feed poorly<br>4. Has fever<br>5. Eye ball sunken<br>6. Increased thirsty<br>7. Irritability or restlessness<br>8. Increased frequency of diarrhea<br>8. Only diarrhea<br>9. Someone (Husband, HEW, neighbor) told to take the child to health facility<br>10. Other (specify)……	
304	Frequency of stool per day	------------ In days.	
305	Type of diarrhea	1. Bloody<br>2. Mucoid<br>3. Watery<br>4. Other (specify)……….	
306	Who was decided to take the child for medical treatment?	1. My self<br>2. Fathers<br>3. Grand parents<br>4. Other (specify)...................	
307	Did the child encounter diarrhea before in the last six month?	1. Yes 2. No	
308	If yes to Q307, did you visit health facility?	1. Yes 2. No	**If No, skip to Q311**
309	If yes to Q308, did that visit help you for your today's visit?	1. Yes<br>2. No	
310	If Yes to Q309, how?	1. Counseled about the importance of visiting health facility for diarrhea<br>2. Told the danger of not visiting health facility for diarrhea<br>3. Satisfied with the treatment given<br>4.Satisfied with the respect given<br>5.Satisfied with examination of child<br>6. Other (specify)…………….	
311	If No to Q308, how was the child cured?	1. Take to traditional treatment<br>2. Self-treatment at home<br>3. Treat the child with drug buying from pharmacies or drug sellers without prescriptions<br>4. Treat with holy water<br>5. Resolved by it self<br>6. Other (specify)………..	
312	Is there child died of diarrhea in	1. Yes	

	the family or neighbor before?	2. No	

Section 4: Promptness of treatment seeking for diarrhea in children

401	How long your child had illness before seeking medical care?	1. within < 24 hours after the onset of diarrhea<br>2. After 24-48 hours of onset of diarrhea<br>3. After 48-72 hrs of onset of diarrhea<br>4. After 3 to 7 days onset of diarrhea<br>5. After seven days of onset of diarrhea	**Skip if within one day to Q405**
402	If after one day of onset of diarrhea, What were the main reasons you were not seek medical care immediately?	1. The disease resolve by itself over time<br>2. The disease resolved by itself before on the child or other child<br>3. Transportation difficulties.<br>4. Cost of medical care<br>5. No trust on health providers' competency.<br>6. Fear that S/he don't get immediate care or several waiting time in facility<br>7. Giving traditional medication at home<br>8. Buy medication from drug vendors<br>9. No treatment for the sickness<br>10. Lack of time<br>11. Shortage/lack of money<br>12. Illness was mild<br>13. Other (specify)………..	
403	If you select traditional healer, what is the most important reason for sorting traditional healers	1. Don't get cure from medical care<br>2. They do not charge to much<br>3. They are respectful<br>4. There is no long waiting time<br>5. Treatment is effective<br>6. Maintain confidentiality<br>7. Maintain privacy<br>8. Because, family recommended it<br>9. Because, they are near<br>10. Other (specify)…….	
404	If you select private drug vendors, what is the most important reason for sorting drug vendors	1. They do not charge to much<br>2. They are respectful<br>3. There is no long waiting time<br>4. Treatment is effective<br>5. Maintain confidentiality<br>6. Maintain privacy<br>7. Because, family recommended it<br>8. Because, they are near<br>9. Other (specify)…….	
405	If within one day of the onset of	1. Previous experience of the delay result in severity	

	diarrhea, what makes you urge to seek medical care early?	2.Symptoms worsen<br>3. Get the information about importance of early seeking for diarrhea.<br>4. Other (specify)……	
406	If you get information about the importance of early treatment seeking for childhood diarrhea, what is your source of information?	1. Health workers<br>2.HEWs through training or health education<br>3. During community conversation<br>4. Neighbors<br>5. Media (Television, radio)<br>5. Other (specify)…………….	
